# Coerced Contracting is Not a Reasonable Solution to Balance Billing

**DOI:** 10.5811/westjem.2016.11.32829

**Published:** 2017-01-19

**Authors:** R. Myles Riner

Regarding the article on access to in-network emergency physicians, the authors conclude that a solution to the lack of access to in-network emergency physicians at many hospitals may be to require plans to contract with these physicians at hospitals that are in-network with the plan (if I understand their approach correctly). Though this mandate might be helpful in some cases, it is just as likely to increase the incidence of coercive contracting, where the plan puts pressure on a hospital in their network to force the emergency physician group at the hospital to accept deeply discounted rates from the plan, or be replaced by another group that will. A better solution would be for plans to be required to pay out-of-network emergency physicians (and on-call specialists) based on a benefit for out-of-network services that is a commercial market-based representation of the reasonable value of these services. Some percentile of usual and customary charges, using a database like the one established by FAIR Health, would provide such a reasonable value standard, while limiting outlier charges that are excessive and unreasonable. This approach is predicated on the idea that most physicians’ charges are reasonable, are designed to address practice costs and overhead, allow these physicians to meet their EMTALA mission to provide care to all, regardless of insurance status or ability to pay, and are subject to the pressures of the market for these services. This in turn would encourage plans to negotiate fairly with emergency physician groups, and not just take advantage of the EMTALA obligation or coercive contracting. It would also eliminate the need for so-called surprise balance billing.

