# Supplementary material for: Coerced Contracting is Not a Reasonable Solution to Balance Billing
Source: West J Emerg Med. 2017 Jan 19;18(2):327. doi: 10.5811/westjem.2016.11.32829 (PMC5321263; doi:10.5811/westjem.2016.11.32829)
Supplement: Related texts

# In reply to: Coerced Contracting is Not a Reasonable Solution to Balance Billing

**Published:** 2017-01-19

**Authors:** Ali S. Raja, Stephen Dorner

**Affiliations:** * Massachusetts General Hospital, Department of Emergency Medicine, Boston, Massachusetts; † Harvard Medical School, Department of Emergency Medicine and Radiology, Boston, Massachusetts

We appreciate the letter to the editor and are pleased to respond to comments regarding our article on in-network access to emergency physicians. In our article, we highlighted that the present methods used by CMS to determine network adequacy for physicians in most medical specialties are not applied to emergency medicine. Rather, CMS enforces minimum payment thresholds for out-of-network emergency medical care. That threshold, known as the “greatest of three,” is the greatest of the following: the plan’s median payment amount for in-network providers, a payment based on the usual methods the plan uses to determine payments for other out-of-network services, or the amount that Medicare would pay for those services. Following this model (and the data that we presented regarding in-network physicians), we concluded that the present regulatory structure disincentivizes the formation of adequate emergency physician networks and therein incentivizes the practice of balance billing as physicians seek to compensate for the out-of-network care they provide.

We proposed that - in lieu of applying network adequacy standards to emergency physicians – and rather than defaulting to the present out-of-network payment thresholds, all emergency physicians should be paid an in-network rate negotiated with insurers. The letter to the editor suggests that it would be better to use a standard threshold of usual, customary, and reasonable (UCR) charges set by the market. However, we identify several issues with this proposal.

UCR charges are typically the highest of the “greatest of three,” because they are the product of both (lower) in-network and (higher) out-of-network rates. As such, the adoption of a system defaulting to UCR charges would reasonably disincentivize emergency physicians from entering networks in favor of the higher out-of-network UCR charge. Furthermore, in defaulting to UCR charges, the practice of balance billing would become unnecessary, eliminating one of the incentives of coercive contracting.

However, defaulting to UCR charges would also change the incentives for emergency physicians to enter networks in the first place, as out-of-network emergency physicians would receive a higher rate by default. This may in turn result in a snowball effect wherein more physicians remain out of network, driving up UCR charges. It is foreseeable that such a scenario would incentivize carriers to actually increase the practice of coercive contracting so as to avoid paying higher UCR charges. That could, in turn, lead to an ultimate loss in physician reimbursements - even below present in-network rates.

Another issue with UCR charges is the present lack of transparency in their calculation. As recently as 2010, UnitedHealthcare subsidiary Ingenix was found guilty of manipulating data to underpay physicians, resulting in a fine of $300 million and the creation of a third-party, nonprofit database for charge data called FAIR health. In May 2016, after our article was published, ACEP filed a lawsuit against HHS for a similar claim, asserting that the “greatest of three” defaults lack transparency. It is unclear what will come of the suit, but the issues with UCR charges remain.
